# The Effect of *Momordica charantia* in the Treatment of Diabetes Mellitus: A Review

**DOI:** 10.1155/2021/3796265

**Published:** 2021-01-16

**Authors:** Zhuo Liu, Jing Gong, Wenya Huang, Fuer Lu, Hui Dong

**Affiliations:** ^1^Grade 2016 of Integrated Traditional Chinese and Western Clinical Medicine, Huazhong University of Science and Technology, Wuhan, China; ^2^Department of Integrated Traditional Chinese and Western Medicine, Tongji Hospital, Tongji Medical College, Huazhong University of Science and Technology, Wuhan, China; ^3^Institute of Integrated Traditional Chinese and Western Medicine, Tongji Hospital, Tongji Medical College, Huazhong University of Science and Technology, Wuhan, China

## Abstract

In recent years, many studies of *Momordica charantia* (MC) in the treatment of diabetes mellitus (DM) and its complications have been reported. This article reviewed the effect and mechanism of MC against diabetes, including the results from in vitro and in vivo experiments and clinical trials. The common side effects of MC were also summarized. We hope that it might open up new ideas for further mechanism exploration and clinical application as well as provide a scientific theoretical basis for the development of drugs or foods derived from MC.

## 1. Introduction

Diabetes is a metabolic disease with typical hyperglycemia manifestations, such as polydipsia, polyphagia, polyuria, impaired vision and body weight [1]. With the increase of high-caloric dietary intake and sedentary lifestyle, the number of diabetic patients has increased dramatically. In light of statistics from the World Health Organization in 2016, 422 million people had diabetes in 2014, and the incidence has risen substantially since 1980 [2]. It is no longer particularly and commonly seen in affluent areas, as the prevalence in relatively backward areas is rising even faster [2]. Diabetes is generally divided into four categories: type 1 diabetes (T1DM) (autoimmune *β*-cell destruction usually accompanied by absolute insulin deficiency), type 2 diabetes (T2DM) (a gradual decrease in *β*-cell insulin secretion), gestational diabetes, and specific types of diabetes [3]. It is a chronic disease which can damage the heart, blood vessels [4], ocular surface [5], nerves [6], and musculoskeletal system [7] gradually and has become the leading cause of kidney failure [8] and blindness [9]. Insulin injection and oral hypoglycemic agents are always used to reduce blood glucose levels. Besides, lifestyle management is also recommended fundamentally. However, because of the poor medication compliance [10] and limited access of large populations to conventional antidiabetic drugs, together with the inevitable side effects and resistance of western medicine [11], patients have always been trying to find some effective natural plants, for instance, Indian Ayurvedic medicine, African traditional medicine, Japanese Kampo medicine, and Chinese herbs [12].

## 2. Overview of *Momordica charantia* L

*Momordica charantia* L., also known as bitter gourd, bitter melon, or karela, is an annual climbing plant of the family Cucurbitaceae. It is native to East India and is widely grown and eaten in tropical, subtropical, and temperate regions at present. The vegetable is light green with a long cone shape; it tastes bitter but is popular for its various benefits [13]. There are many nutrients in this plant [14, 15]. With the deepening of research on MC, numerous phytochemicals have been discovered, including saponins, polysaccharides, triterpenes, proteins, vitamins, minerals, flavonoids, ascorbic acid, and steroids [16]. Besides, multiple biological characteristics have also been confirmed, such as antioxidant, hypoglycemic, antitumor, antibacterial [17], skin care [18], anthelmintic, neuroprotective, anti-inflammatory, antiviral, immunomodulatory, wound healing promoting, antimutagenic, antiulcer, liver protection, and antiobesity activities [16, 19–21]. It commonly serves as four dosage forms: fruit juice, freeze-dried powder, entire fruit, or capsule, and the preparations are mainly crude extracts (usually extracted with water, ethanol, or methanol) and effective monomer components extracted from its fruit, seeds, or leaves [22]. As a multifunctional vegetable, MC plays a vital role in traditional medicine in China, India [13], Mauritius [23], Turkey [24], and other Asian and African regions [25]. Nowadays, the research on various potentials of MC is in full swing, and the antidiabetic effect is of particular concerns. In this review, the reports on the treatment of diabetes by MC were summarized.

## 3. Antidiabetic Efficacy and Mechanism of *Momordica charantia*

### 3.1. Enhancing Glucose Uptake, Consumption, and Utilization

#### 3.1.1. Cell Experiments

Cytological research concentrates on studying the influence of MC on insulin signaling pathways of insulin-targeted cells such as hepatocytes, skeletal muscle cells, and adipocytes. The mechanisms of MC concentrate on improving insulin resistance through enhancing glucose uptake, consumption, and utilization.

A novel insulin receptor-binding protein from *Momordica charantia* (mcIRBP) was separated from MC. A further study identified mcIRBP-9 and confirmed the gastric resistance and hypoglycemic activity of the peptides [26]. The protein physically interacts with the insulin receptor (IR) through the binding sites different from that of insulin, and it shows a synergistic effect with insulin, enhancing the kinase activity of IR by 5.87 ± 0.45 times and increasing the amount of phospho-IR protein by 1.31 ± 0.03 times. All in all, mcIRBP motivated the glucose consumption of cells by 1.36 ± 0.12 times via affecting IR/phosphoinositide-3-kinase (PI3K)/protein kinase B (Akt) pathways and consequently promoting translocation of glucose transporter 4 (GLUT4) [27].

The research on cucurbitane-type glycosides isolated from MC shows that the compounds can increase the uptake of glucose by the C2C12 myoblasts [17], and they exert antidiabetic effect by behaving as a stronger adenosine 5′-monophosphate-activated protein kinase (AMPK) activator than troglitazone [28]. However, one study demonstrates that the cucurbitane-type triterpenoids can stimulate glucose utilization in C2C12 myotubes through insulin receptor substrate-1 (IRS-1) signaling pathway in skeletal muscle instead of adipose tissue and liver tissue [29]. Besides, MC juice can stimulate glucose elimination in L6 myotubes [30], and the protein extract of MC has similar effect on C2C12 myocytes and 3T3-L1 adipocytes as well [31]. Interestingly, it was observed that a standardized MC fruit ethanolic extract improved glucose uptake of hemidiaphragm in vitro [32], and a further study indicated that MC juice could do it in the presence and absence of insulin [33]. Moreover, it has been reported that bitter gourd methanol extract can promote glucose uptake of insulin-resistant FL83B cells [28]. Therefore, we speculate that MC may have a hypoglycemic effect in a non-insulin-dependent manner and show an insulin signaling pathway-enhancing effect.

**Figure 1:**
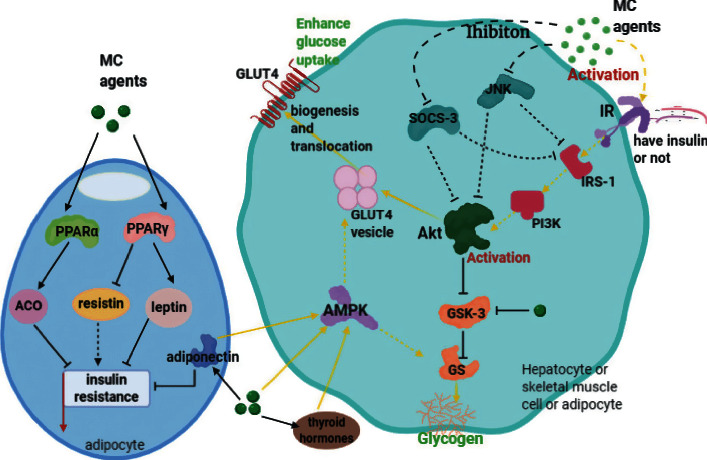
Mechanisms of MC in enhancing glucose uptake and alleviating insulin resistance. MC agents function by inhibiting the expression of SOCS-3 and JNK, activating IRS-1/PI3K/Akt pathway directly or indirectly, and thus promoting the biogenesis and translocation of GLUT4 and suppressing GSK-3. GSK-3 can also be blocked through connecting with MC agents. The activity of AMPK influenced by increased thyroid hormones, adiponectin, and MC agents diametrically also plays a role in stimulating GLUT4 and GS. Besides, MC stimulates adiponectin and the PPAR-*α*/PPAR-*γ* pathway that can upregulate ACO and leptin and downregulate resistin to alleviate insulin resistance.

Additionally, it has been recorded in the literature that three insulin-like active compounds from bitter gourd can block the active site of glycogen synthase kinase 3 (GSK-3) by combining with it, thus reducing the inactivation of glycogen synthase (GS) and further restoring glycogen content [34] (Figure 1).

#### 3.1.2. Animal Experiment

Animal experiments mostly focus on the therapeutic effect of MC on T2DM through ameliorating insulin signal transduction disorder, while there are few documents confirming the impact of MC on T1DM. In the treatment of diabetic rats induced by high-fat diet (HFD) and streptozocin (STZ), the ethanol extract functions by downregulating the expression of suppressors-of-cytokine-signaling 3 (SOCS-3) and c-Jun N-terminal kinase (JNK), improving IRS-1/PI3K signal transduction, and upregulating the expression of Akt-2 and GLUT-4 in skeletal muscle and liver tissues, as well as increasing liver glycogen content [35] (Figure 1). In diabetic rats fed with HFD, MC powder motivates insulin signaling through elevating phospho-IRS-1 (Tyr612) and phospho-Akt (Ser473) in muscle and liver, along with lowering phospho-JNK (Thr183/Tyr185) in muscle, liver, and epididymal adipose [36]. Moreover, it is found that MC juice can increase the glucose uptake of skeletal muscle via PI3K pathway [30]. Aqueous extract is able to improve the protein expression of IRS-1 in liver tissue and GLUT4 in skeletal muscle so as to enhance glucose utilization in HFD-induced T2DM KK/HI mice, but not in STZ-induced T1DM ICR mice [37]. In 2006, cucurbitane triterpenoids isolated from MC were first proved to have hypoglycemic activity in male ddY diabetic mice [38]. Further studies on STZ- and alloxan- (ALX-) induced DM mice models have revealed that such compounds increase the activation of IRS-1/PI3K/Akt pathway, leading to translocation of GLUT4 and suppression of GSK-3*β*, thereby stimulating glucose uptake by skeletal muscle [29] and other tissues, increasing liver glycogen content at the same time [39]. As we have mentioned above, MC contains a novel insulin receptor-binding protein, mcIRBP, which can combine with IR and activate downstream signal transduction pathways [27]. An experiment on T1DM mice indicated that oral administration of mcIRBP-9 for 30 days resulted in the clearance of blood glucose and a distinct reduction in hemoglobin A1c (HbA1c) levels [26].

Other metabolic signaling pathways involved in the hypoglycemic effect of MC were also investigated. Research on T2DM KK-Ay mouse model suggests that water extract of MC affects glucose utilization by activating AMPK in muscle tissue [40]. Studies on obese rats have shown that MC enhances the activity of AMPK by stimulating the production and function of thyroid hormones and adiponectin, bringing about the increase and translocation of GLUT4 [41]; bitter melon juice exhibits the function of alleviating insulin resistance, and the mechanism may involve the upregulation of leptin and adiponectin [42] (Figure 1). In ALX-induced DM mice model, saponins from MC have a significant effect on activating AMPK, which promotes glycogen synthesis [43] via glycogen synthase [44]; cucurbitane triterpenoids can improve cellular uptake of glucose via upregulating the transcription levels of AMPK-*α*1 and GLUT4 [39]. In addition, in vivo and in vitro research has found that MC extract can influence peroxisome proliferator-activated receptor (PPAR) PPAR*α*/PPAR*γ* and further regulate acyl-CoA oxidase (ACO) [45, 46], leptin, and resistin, increasing glucose utilization of adipocytes in HFD mice consequently [47] (Figure 1).

### 3.2. Delaying Glucose Absorption

#### 3.2.1. Cell Experiment

An experiment was performed to assess the inhibitory activity of protein extracts from two kinds of bitter melon on *α*-amylase and *α*-glucosidase, and it showed that their inhibition rate (66% to 69%) and IC50 (0.26 to 0.29 mg) were comparable to acarbose [48]. It has been proved that the inhibitory activity of MC ethanol extract on pancreatic lipase and *α*-glucosidase is performed in a dose-dependent manner [49]. Moreover, the suppression of the ethyl acetate fraction of MC extract on pancreatic lipase is positively correlated with its phenol content [50]. Momordicin, a compound identified from MC, acted against *α*-amylase with IC50 of 15.86 *μ*g/ml [51]. An acidic and branched heteropolysaccharide obtained from MC was found to have 89.1% *α*-amylase inhibitory property [52]. The inhibitory activity of cucurbitane-type compounds in *α*-glucosidase and *α*-amylase has also been observed [53, 54]. The inhibitory properties of compounds 1–7 isolated from MC on *α*-glucosidase and *α*-amylase are between 35% and 70%; besides, gentisic acid 5-O-*β*-d-xyloside and momordicoside G showed the strongest effect on *α*-glucosidase (56.4%) and *α*-amylase (70.5%), respectively [55]. Total anthocyanins from bitter melon [56], oil, and polypeptide-k extracted from MC seeds [57] can also contribute to the inhibitory effect of MC on *α*-glucosidase and *α*-amylase. Besides, it is suggested that fermentation of MC juice with lactic acid bacterium improves its inhibition of *α*-glucosidase activity [58], providing a new idea for researchers to acquire more efficient components from bitter melon in the future.

#### 3.2.2. Animal Experiment

**Figure 2:**
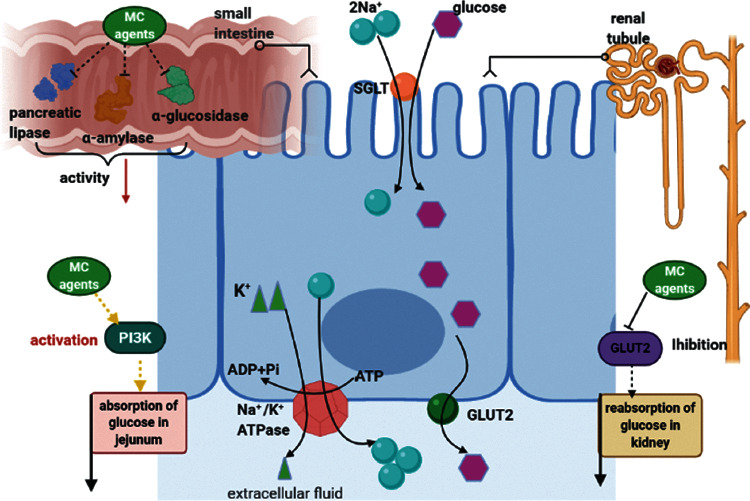
Mechanisms of MC in delaying glucose absorption. In the small intestine, MC agents delay the digestion and absorption of food because of the inhibitory activity in *α*-amylase, pancreatic lipase, and *α*-glucosidase. MC agents also activate PI3K, which is associated with less glucose absorption in the jejunum epithelium, and suppress GLUT2, which is associated with decreased reabsorption of glucose in the kidney.

In STZ-induced diabetic rats, MC juice reduces the Na^+^- and K^+^-dependent absorption of glucose in jejunal brush border membrane vesicles by affecting PI3K [30]; MC powder [59] and fresh fruit [60] when given orally affect glucose absorption due to dietary fiber. Furthermore, MC powder can lower the expression of GLUT2, thus reducing the reabsorption of glucose by the kidneys and increasing the excretion of urine sugar [61] (Figure 2).

### 3.3. Inhibiting Gluconeogenesis and Glycogenolysis

**Figure 3:**
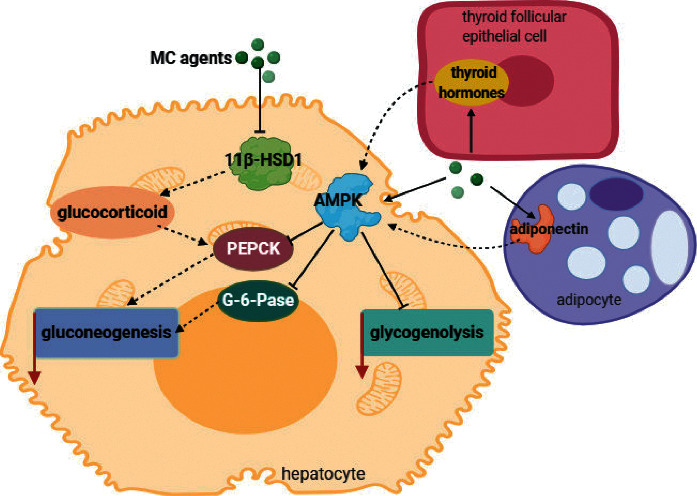
Mechanisms of MC in inhibiting gluconeogenesis and glycogenolysis. MC agents can activate AMPK directly and indirectly to reduce glycogenolysis and PEPCK together with G-6-Pase, both of which are key enzymes of gluconeogenesis. At the same time, MC has the ability to suppress 11*β*-HSD1, leading to the reduction of glucocorticoids and further reducing PEPCK to inhibit gluconeogenesis.

According to reports, MC extract treatment can inhibit glycogenolysis in liver slices in vitro and in liver tissue in ALX-induced diabetic rats [32]. Meanwhile, saponins from MC have the ability to promote AMPK phosphorylation, thereby preventing gluconeogenesis in the liver of diabetic mice [43]. Increased activation of fat 11beta-hydroxysteroid dehydrogenase type 1 (11*β*-HSD1) is currently recognized as an important cause of obesity and type 2 diabetes epidemics. An experiment in vitro with respect to the effect of MC extract on 11*β*-HSD1 indicates that the activity of 11*β*-HSD1 is inhibited in dose-dependent and selective manner, resulting in the decrease of intracellular glucocorticoids concentrations, and further inhibits phosphoenolpyruvate carboxykinase (PEPCK) [62]. It is also demonstrated that MC ethanol extract can inhibit hepatic glycosylation in HFD mice, and the effect is attributed to less expression of PEPCK and glucose-6-phosphatase (G-6-Pase), owing to the increase of AMPK phosphorylation and decrease of liver 11*β*-HSD1 [63] (Figure 3).

### 3.4. Protecting Islet *β* Cells

#### 3.4.1. Cell Experiment

Four new cucurbitane-type triterpenes derived from MC show a protective effect on H_2_O_2_-damaged pancreatic cells; in particular, the protection ratio of compounds 2 and 3 reached up to 94.85% and 92.85%, separately. It is also found that the saponin-rich part of MC motivated insulin secretion in pancreatic *β* cells of MIN6 in a concentration-dependent manner [64]. An experiment on the treatment of high glucose-treated RIN-m5F pancreatic *β* cells with the aqueous extract of MC shows that the survival rate of cells was significantly higher than that of untreated glucotoxic cells after 24 hours, but no protective effect was detected after 72 hours [37]. The underlying mechanism of this beneficial effect is still unknown. One study believes that it is attributed to the increase in the secretion of glucagon-like peptide-1 (GLP-1) which may help toward beta-cell proliferation and insulin secretion. Water extract of bitter melon stimulates the secretion of GLP-1 in the mouse enteroendocrine cell line STC-1 in a dose-dependent way. In addition, it works through certain bitter taste receptors (type 2) (TAS2R) and/or phospholipase C *β*2-signaling pathway (PLC-*β*2), and two cucurbitane triterpenoids isolated from MC display high efficacy [65]. Besides, it is recorded that excessive glucocorticoids regulated by 11*β*-HSD1 are involved in the beta-cell damage and impair glucose-stimulated insulin secretion (GSIS) [66–68], while MC extracts can inhibit the activity of 11*β*-HSD1 significantly [62].

#### 3.4.2. Animal Experiment

**Figure 4:**
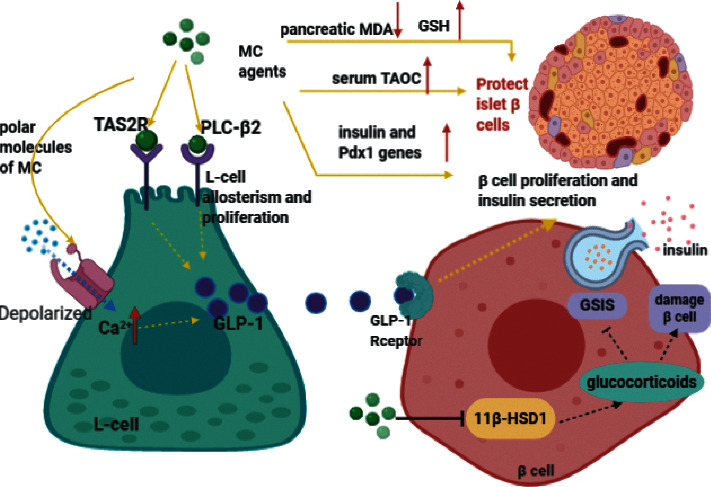
Mechanisms of MC in protecting islet *β* cells. MC agents promote L-cell allosterism and proliferation through TAS2R or PLC-*β*2 and depolarize L cells to elevate the secretion of GLP-1, which can improve *β*-cell proliferation and insulin secretion. In addition, MC agents inhibit 11*β*-HSD1, decreasing local glucocorticoid aggregation, to ameliorate GSIS and *β* cells. On the other hand, MC agents upregulate insulin and Pdx1 genes, raise serum TAOC, pancreatic GSH, and lower pancreatic MDA to mitigate *β*-cell damage and enhance pancreatic function.

In STZ-induced diabetic mice, it has been confirmed that a polysaccharide isolated from MC (MCPIIa) [69] and *Momordica charantia* polysaccharide-chromium (III) complex (MCPIIaC) [70] are effective in protecting pancreatic cells and increasing plasma insulin; selenylated polysaccharide from MC can also increase insulin level [71]. A series of experiments on STZ-induced diabetic rats further confirmed this activity and investigated the underlying mechanism. MC juice treatment can raise the quantity of *β* cells by promoting the renewal or recovery of destroyed *β* cells [72], and it significantly improves insulin levels as well as the amount of insulin-positive cells per islet [30]. MC juice taken orally can also improve the histopathological changes of pancreas and *β* cell function percent in the way of reducing pancreatic malondialdehyde (MDA) content, increasing serum total antioxidant capacity (TAOC) and pancreatic glutathione (GSH) levels [33]. The results are consistent with the findings of Wang et al. who reported that *Momordica charantia* polysaccharide (MCP) distinctly improved antioxidant capacity by increasing superoxide dismutase (SOD) content and reducing MDA content, and therefore it mitigated the damage of pancreas caused by STZ and repaired pancreatic *β* cells [43]. Besides, treatment with ethanolic extract of MC almost doubles the total quantity and area of *β* cells and keeps the quantity of *β*-cell insulin granules equivalent to that of nondiabetic group [73]. Subchronic studies of ALX-induced diabetic rats have similar findings. A standardized ethanolic extract of MC is valid for restoring the altered histological architecture and enhancing insulin secretion of the islets of Langerhans [32]. The hypoglycemic effects of acetone extract [74] and alcoholic extract [75] have also been found to be closely related to the promotion of *β*-cell regeneration (Figure 4).

In addition, experiments pertaining to the mechanism of MC on activating *β* cells have other discoveries. An article states that MC powder can improve pancreatic function by activating pancreatic *β* cells, evidently increasing the expression of insulin and pancreatic duodenal homeobox factors-1 (Pdx1) genes [61]. As we know, Pdx1 is beneficial to the regulation of pancreatic development and stimulation of *β* cell growth [76]. One study in healthy and diabetic Wistar rats illustrates that the effect of MC aqueous extract on the elevation of *β* cell proliferation and insulin secretion is related to the elevation of GLP-1 level, and it may result from the enteroendocrine L-cell allosterism and proliferation; besides, the polar molecules of MC depolarize L cells by raising intracellular Ca^2+^ concentration, which in turn strengthens the release of GLP-1 [77]. Another study suggests that water extract of bitter gourd stimulates GLP-1 and insulin secretion in mice, and the hypoglycemic effect can be revoked by GLP-1 receptor antagonist [65]. Subcutaneous injection with protein extract from MC fruit pulp can also lower plasma glucose and elevate insulin concentration via both insulin secretagogue and insulinomimetic activities, and this is further proved through pancreatic perfusion in situ [31].

All in all, the *β*-cell protection effect of MC is related to pancreatic MDA, GSH and serum TAOC, upregulation of insulin and Pdx1 genes, increased GLP-1 secretion via TAS2R, PLC-*β*2, and intracellular Ca^2+^ concentration, as well as the inhibition of 11*β*-HSD1 (Figure 4).

### 3.5. Antioxidation

#### 3.5.1. Cell Experiment

It is shown that aqueous extracts of MC pulp as well as flesh extracts are capable of preventing the accumulation of crosslinked advanced glycation end products (AGEs) and carboxymethyl lysine (CML), which is probably owing to the antioxidant characteristic, especially the total phenolic content of the extracts [78]. Flavonoids abstracted from ethanol-modified SC-CO_2_ extraction method have great ability to clear 1,1-diphenyl-2-picrylhydrazyl (DPPH) free radical, which reached up to 96.14 ± 1.02%, corresponding to the clearance rate of ascorbic acid at 1.2 mg/mL [79]. An acid branched heteropolysaccharide derived from MC displayed free radical scavenging activity (31.9%) and ferric reducing antioxidant power (FRAP) (0.95 mM) [52]. It is also found that chemical modification without changing the overall properties can effectively optimize the activity of MCP. Sulfated MCP exhibits stronger effect on anti-lipid peroxidation and superoxide anion (O^2−^) scavenging. On the other hand, phosphorylated MCP shows higher antioxidant activity on both ability to scavenge hydroxyl radical (HO·), O^2−^, and DPPH radical and the antilipid peroxidation and reduction ability [80]. It has been reported that choosing opportune harvest time can significantly increase the content of total anthocyanins in MC, contributing to the higher free radical scavenging and antidiabetic activities of it [56].

#### 3.5.2. Animal Experiment

In STZ-induced diabetic mice, MCPIIaC strengthens the antioxidant enzyme defense system and weakens peroxidation of lipids in a dose-related manner, which indicates that the antidiabetic effect of MCPIIaC may be relevant to its antiglycation ability [70]. Selenylated MCP reinforces its hypoglycemic activity by increasing antioxidant enzyme activity [71]. MCP orally restores TAOC by improving the level of SOD and declining the level of MDA [43]. It is also shown that MCP treatment in diabetic rats can significantly increase GSH, SOD, and catalase (CAT) levels via heme oxygenase-1 (HO-1)/nuclear factor erythroid 2-related factor 2 (Nrf2) pathway, while it can decrease MDA in diabetic kidneys [81]. In addition, in high-fat-induced obese rats, allosteric MCP through *Momordica charantia* fermentation decreases the lipid and oxidative stress level, resulting in the remarkable reduction of insulin resistance [82].

**Figure 5:**
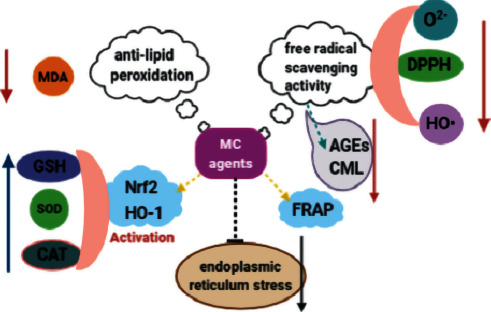
Mechanisms of MC in antioxidation. MC agents exhibit extraordinary O^2−^, HO·, DPPH, and other free radicals scavenging activity to lighten oxidative stress, resulting in the less production of MDA, AGEs, and CML. MC agents also increase the content of GSH, SOD, and CAT by activating HO-1/Nrf2 pathway. Besides, MC agents have FRAP and capability of ameliorating endoplasmic reticulum stress.

What is more, it has been recorded in the literature that not only oxidative stress but also endoplasmic reticulum stress plays crucial roles in the pathogenesis of diabetes [83]. An article demonstrates that MC treats diabetes by ameliorating endoplasmic reticulum stress and oxidative stress [84]. We can see the different aspects of antioxidant effect of MC more clearly in Figure 5.

### 3.6. Anti-Inflammation

#### 3.6.1. Cell Experiment

**Figure 6:**
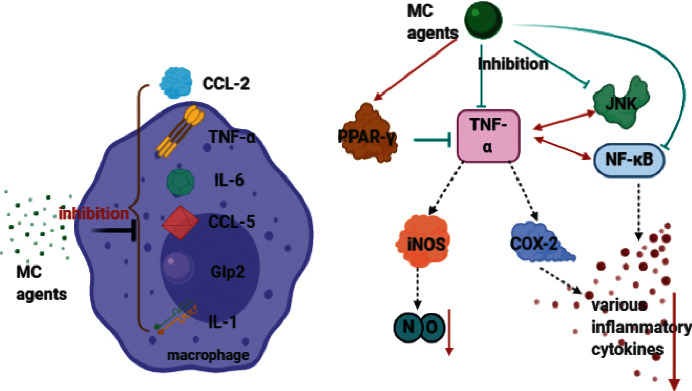
Mechanisms of MC in anti-inflammation. MC agents can inhibit TNF-*α* straightly or in the way of stimulating PPAR-*γ* and suppressing JNK, to lessen the expression of iNOS and COX-2, thus reducing NO and multiple inflammatory cytokines. MC agents can also inhibit NF-*κ*B, which is concerned with TNF-*α*, to lower the production of various inflammatory cytokines. Besides, the expression of IL-1, IL-6, CCL-5, G1p2, and CCL-2 can be inhibited in the macrophages.

Type 2 diabetes is considered to have a chronic low-grade inflammation state, and the increase of inflammatory factors may affect insulin signal transduction, which is related to the formation of insulin resistance [85]. Many reports have shown that gentisic acid 5-O-*β*-d-xyloside [55], cucurbit type triterpenes [54] identified from MC, and butanol fraction of bitter gourd extract have obvious effects on inhibiting the expression of tumor necrosis factor–*α* (TNF-*α*), IL-1*α*, IL-1*β*, IL-6, G1p2 (gene symbol of interferon,  *α*-inducible protein), chemokine  (C−C  motif)  ligand 5 (CCL5), and other inflammatory genes in lipopolysaccharide-induced RAW264.7 cell [86], with a significant decrease of nitric oxide (NO) in hepatocytes [87]. The anti-inflammatory effect of cucurbit type triterpenes is also confirmed in HepG2 cells. Seventeen cucurbitane-type triterpene glycosides (1–17) isolated from MC evidently inhibit the activity of NF-*κ*B mediated by TNF-*α*, and some also suppress inducible nitric oxide synthase (iNOS) and cyclooxygenase (COX-2) expressions and stimulate PPAR-*γ* significantly [88, 89] (Figure 6).

#### 3.6.2. Animal Experiment

In HFD OLETF rats, MC powder significantly decreases proinflammatory cytokines (IL-6, TNF-*α*, and CCL2) in liver, muscle, and epididymal fat by preventing the phosphorylation of JNK and nuclear translocation of NF-*κ*B, thus reversing the inflammation-induced changes in IRS-1 and Akt, which ameliorate glucose tolerance and insulin sensitivity [36]. On the other hand, in HFD obese mice, MC powder treatment exhibits an activity against the infiltration of obesity-related macrophage and mast cell and the expression of inflammatory cytokines in adipose tissue [90]. The ethyl acetate-soluble fraction of MC extract, containing cucurbitacin B, markedly suppresses the expression of IL-1*β* mRNA, TNF-*α* mRNA, and iNOS gene in T2DM ob/ob mouse model [87] (Figure 6). A minority of studies have also found the anti-inflammatory effect of MC in T1DM. It has been proved that this effect of MC juice is associated with the anti-inflammatory and immunosuppressive activities of T-helper 2 cell. The juice promotes the anti-inflammatory phenotype of Th2 by decreasing Th1 cytokines (IL-2, interferon-*γ*) and increasing Th2 cytokines (IL-4) and IL-10 (regulatory cytokines). It exerts anti-inflammatory effects by reducing proinflammatory cytokines (IL-1, IL-6, TNF-*α*, and IL-7) and elevating anti-inflammatory cytokines (TGF-*β*, IL-10). As a result, MC juice prevents the effect of autoreactive T cells on *β* cells in T1DM rats [91, 92].

Additionally, other mechanisms underlying the antidiabetic effect of MC have been investigated, especially the role of intestinal microbiota disorders in driving type 2 diabetes [93]. In STZ-induced diabetic rats, dried bitter gourd powder feeding can improve the diabetic status of rats, which may be attributed to high fiber content [59], especially pectin, a kind of soluble dietary fiber in fresh fruit. This fiber can regulate intestinal flora and increase intestinal SCFA concentration and fecal cholesterol secretion [60]. Studies also demonstrate that MC fermentation with *Lactobacillus plantarum* NCU116 optimizes the structure of polysaccharides, leading to the improvement of intestinal flora and colon SCFA production, which enhances its antidiabetic effect [82]. Another report reveals that MC preparation can alter the proportion of specific bacterial flora and influence the intestinal epithelial barrier and intestinal mucosal immunity, thereby adjusting inflammation levels and eventually mitigating insulin resistance and diabetes in diabetic rats. The mechanism is named as “Bacteria-Mucosal Immunity-Inflammation-Diabetes Axis” [94], which may be a potential insight that deserves to be explored in depth.

Over and above that, in the research carried out by Ebrahim et al., it is found that the bitter gourd fruit has the highest extractable zinc concentration, and zinc has the functions of insulin simulation and insulin-secretion promotion, as well as regulation of GLUT4 transport and glucose utilization, which conduce the hypoglycemia of MC [95].

Moreover, the hypoglycemic activity of different dosage forms and extraction methods of MC has been evidenced in different animal models. In the ALX-induced diabetic rat model, the water extract of MC is more effective than dried fruit powder. The former also delayed the onset of cataracts, which indicates the adaptability of MC [96]. An experiment, using normal and ALX-induced diabetic rabbits as subjects, demonstrates the significant and consistent hypoglycemic effect of MC [97]. The antihyperglycemic activity of MC peptide and its peptide analog series have been represented in STZ-induced diabetic mice [98], as well as MC water-soluble polysaccharide in ALX-induced diabetic mice [99]. Additionally, it is reported that pretreatment with MC juice succeeds in reversing the pathological damage caused by STZ in diabetic rats via controlling hyperglycemia, hyperlipidemia, and oxidative stress but fails in preventing the occurrence of disease [33].

## 4. Clinical Trials

As the hypoglycemic properties of MC have been generally verified in cell and animal experiments, some small-sample clinical trials have been performed. In 1993, a clinical trial was conducted in 12 male diabetic subjects with mild (postprandial blood sugar of 260 mg%) to severe (postprandial blood sugar of 433 mg%) degree. The result suggests that the hypoglycemic effect of MC is outstanding but cumulative and gradual. MC water extract is much better than dry powder and can delay the onset of cataracts and other secondary complications [96]. MC homogenized aqueous suspension treatment in 100 moderate non-insulin-dependent diabetic mellitus (NIDDM) patients results in a notable decline in postprandial and fasting blood glucose (FBG) with 86%, and 5 cases only show the reduction in FBG [100]. In 2004, Chinese researchers made kugua jiangtang capsule with water-soluble active ingredients of bitter gourd and applied it to 35 elderly male patients with T2DM. After one course treatment (30 days), both the blood sugar and lipid had a remarkable improvement [101]. In the same period, patients with T2DM in India (50–65 years old) were treated with MC extract (CCl4 + C6H6 MC soft extract) plus a half-dose oral hypoglycemic agent (metformin, glibenclamide, or a combination of both) for 7 days, and ultimately the hypoglycemic effect was greater than that of full-dose hypoglycemic drugs, indicating that MC extract has synergy with oral hypoglycemic drugs [102].

A randomized controlled trial (RCT) of MC and rosiglitazone treatment in NIDDM patients reveals that rosiglitazone treatment can elevate the serum sialic acid, which is associated with diabetes and complications, while MC group keeps it comparable to normal subjects [103]. Similarly, a 4-week, double-blind RCT evaluates the efficacy of bitter melon pulp powder and metformin in newly diagnosed T2DM patients, and, conclusively, bitter melon has moderate hypoglycemic effect [104]. Furthermore, a RCT comparing the different effects of bitter melon and glibenclamide concludes that the hypoglycemic effect of bitter melon is inferior to that of glibenclamide, but bitter melon can ameliorate the diabetes-related cardiovascular risk factors more effectively [105]. Another double-blind RCT in 24 T2DM cases illustrates that MC administration for three months lowers the HbA1c, 2 h glucose, glucose AUC, and mass index and elevates the insulin secretion obviously [106].

In 2012, a study focused on 42 metabolic syndrome patients reported that bitter gourd treatment for three months evidently decreased the incidence of metabolic syndrome, which is recognized for predicting T2DM, and the improvement could remain for one month after stopping supplement [107]. An acute experiment in prediabetic adults exhibited that acute intake of bitter melon beverage led to the reduction of postprandial glucose in 50% of the subjects [108]. Next year, a RCT on 52 individuals with prediabetes showed that MC intake dropped the blood glucose in participants with higher baseline FBG levels more significantly [109]. Accordingly, we speculate that the hypoglycemic effect of MC will be more excellent in diabetics. Over and above that, one observational study on incidence and reasons of hypoglycemia in T2DM patients suggests that natural foods accounted for 16.9% of cases, where MC accounted for 54.5%, which also proves its hypoglycemic effect indirectly [110].

However, there are also some studies suspecting the ability of MC to improve the disorder of glucose metabolism. In 2007, a RCT on type 2 diabetics elucidated the issue regarding the hypoglycemic effects of MC for the first time. It is concluded that the difference in average change in HbA1c between MC capsules and placebo was 0.22%, with the hypoglycemic power being lower than 11% [111]. Then, in 2009, a RCT in nondiabetic overweight men demonstrated that acute, single oral administration of MC powder was useless for insulin or blood glucose and energy expenditure [112]. Cochrane systematic reviews in 2010 [113] and 2012 [114] both reported that there was not enough evidence to suggest MC for type 2 diabetes at that time. However, a systematic review and meta-analysis in 2019 drew the opposite conclusion, but the quality of evidence was low [25].

## 5. Effects on Diabetes Complications

### 5.1. Diabetic Microvascular Complications

In STZ-induced T2DM mice, the renoprotective nature of *Momordica* saponins is exerted by improving the level of uric acid and creatinine, while MCP functions mainly through enhancing antioxidant capacity [43]. It is also illustrated that MCP inhibits the exacerbation of diabetic nephropathy by suppressing oxidative stress and regulating HO-1/Nrf2 pathway in STZ-induced diabetic rats [81]. MC extract administration prolongs diabetic nephropathy via protecting kidneys from oxidative damage [115]. On the other hand, bitter gourd feeding controls kidney complications by means of countering the increase in the components of glycoconjugates and the decrease in kidney heparan sulfate during diabetes [116]. Another study observes that MC extract mitigates the apoptosis of retinal ganglion cells in T2DM rats by downregulating Bax or upregulating Bcl-2 mRNA and protein expression levels [117].

### 5.2. Diabetic Macrovascular Complications

Dietary MC freeze-dried powder can optimize multiple lipid parameters in rats fed with or without cholesterol-enriched diets [118], and it may be related to the inhibitory effect of diosgenin, dietary fiber, and phytosterols in MC on cholesterol absorption [60]. Besides, multiple reports have expounded the hypolipidemic activity of *Momordica* saponin [43], MC ethanol extract [32], and MC leaf extract [119]. A clinical RCT also confirms the improvement of dyslipidemia by bitter melon hot water extract [120]. All of the above indicate that MC has antiatherosclerosis potential. In T2DM and related cardiovascular diseases, bitter melon extract enhances cardiac function and mitigates postischemic/reperfused infarct zone, which have correlation with reduced expression of caspase-3 in heart [121]. It is evidenced that MC can be used to treat diabetes-associated cardiac fibrosis because of its inhibitory capacity in fibroblast proliferation and collagen synthesis, which is mediated by suppressing TGF-*β*1/Smad pathway and activating Nrf2 [122]. Polysaccharide from MC is discovered to have powerful angiotensin-converting enzyme inhibitory properties (94.1%) [52], pointing to its application in various cardiovascular complications. What is more, it is reported that nanoparticles of MC extract reduce blood viscosity in hyperglycemic individuals [123] and water extract of MC has inhibitory properties in AGEs accumulation and oxidative stress [78], which are involved in controlling diabetic complications.

In addition, findings suggest that topical application of bitter melon extract can promote the growth of granulation tissue and angiogenesis in diabetic wounds [124]. MC seeds have the potential to mitigate blood lipids and serum uric acid [125].

## 6. Side Effects

Although many research studies show that diverse MC preparations have no significant toxic effects [75, 87, 99], there are quite a few reports illustrating the adverse effects of MC. Researchers discovered *α*-momorcharin, a kind of ribosome-inactivating protein in the seeds of MC, and it stimulated the inflammatory response of human monocytes via activating the IKK/NF-*ĸ*B and JNK pathways, which increased the risk of acquiring inflammation-related diseases for using MC [126]. Toxicological studies of MC on albino rats suggested that subcutaneous injection of MC ethanol extract caused abnormal breathing and heart rate after two hours, and MC juice led to acute poisoning symptoms such as acute convulsions, nervous disorders, jumping, and shortness of breath [127]. Besides, it is found that water extract of immature bitter gourd fruit exhibits its abortion and teratogenic properties when administered to pregnant Sprague Dawley rats [128]. The teratogenicity and cardiotoxicity of MC fruit and seed extracts have been characterized in zebrafish embryos [129]. In female Wistar rats, leaf extracts of MC, oral intake for 30 days, brought about a significant decrease in estrogen and progesterone levels, showing a dose-dependent antifertility effect [130]. However, in male mice, intraperitoneal administration of MC seed extracts displayed an antispermatogenic effect after 48 days [131]. Therefore, MC application should be recommended with caution, especially for the diabetics who are planning to be or have been pregnant.

## 7. Conclusion

In summary, MC is an easily available and cheap vegetable with a broad range of therapeutic activities and insignificant defects. The antidiabetic potential of MC is in line with the general pursuit of a healthy diet and represents a fresh, hopeful method to expand the scope of diabetes treatment. However, due to insufficient clinical evidence and lack of definite curative MC preparations and scientific intervention programs, its application in the field of food and pharmaceuticals is still in its infancy, and it is far from being fully utilized in diabetic patients. Therefore, it is necessary to explore further how to apply the research results to clinical practice reasonably and sustainably to create a better future for people with diabetes.

## Data Availability

No data were used to support this study.

## Abbreviations

MC: *Momordica charantia*
DM: Diabetes mellitus
T1DM: Type 1 diabetes mellitus
T2DM: Type 2 diabetes mellitus
GLUT: Translocation of glucose transporter
IR: Insulin receptor
mcIRBP: Insulin receptor-binding protein from *Momordica charantia*
AMPK: Adenosine 5′-monophosphate-activated protein kinase
IRS-1: Insulin receptor substrate-1
Akt: Protein kinase B
GSK-3: Glycogen synthase kinase 3
PPAR: Peroxisome proliferator-activated receptor
HFD: High-fat diet
STZ: Streptozocin
ALX: Alloxan
HbA1c: Hemoglobin A1c
FBG: Fasting blood glucose
NIDDM: Non-insulin-dependent diabetic mellitus
PEPCK: Phosphoenolpyruvate carboxykinase
G-6-Pase: Glucose-6-phosphatase
ACO: Acyl-CoA oxidase
MCP: *Momordica charantia* polysaccharide
MCPIIaC: *Momordica charantia* polysaccharide-chromium (III) complex
MDA: Malondialdehyde
TAOC: Total antioxidant capacity
GSH: Glutathione
SOD: Superoxide dismutase
AGEs: Advanced glycation end products
CML: Carboxymethyl lysine
DPPH: 1,1-Diphenyl-2-picrylhydrazyl
SOCS-3: Suppressors-of-cytokine-signaling 3
JNK c: Jun N-terminal kinase
11*β*-HSD: 11beta-hydroxysteroid dehydrogenase type 1
GLP-1: Glucagon-like peptide-1
PDX-1: Pancreatic duodenal homeobox factors-1
HO-1: Heme oxygenase-1
Nrf2: Nuclear factor erythroid 2-related factor 2
TNF-*α*: Tumor necrosis factor–*α*
G1p2: Gene symbol of interferon
*α*: Inducible protein
CCL: Chemokine (C−C motif) ligand
NO: Nitric oxide
TGF-*β*: Transforming growth factor-*β*
iNOS: Inducible nitric oxide synthase
COX-2: Cyclooxygenase-2
GS: Glycogen synthase
TAS2R: Bitter taste receptors, type 2
PLC-*β*2: Phospholipase C *β*2-signaling pathway
GSIS: Glucose-stimulated insulin secretion
FRAP: Ferric reducing antioxidant power
O^2-^: Superoxide anion
HO: Hydroxyl radical
CAT: Catalase
SGLT: Sodium glucose cotransporter
Na+/K + ATPase: Na+, K+ stimulated adenosine triphosphatase.
